# Influence of Graphene Oxide on the Ethanol Permeability and Ionic Conductivity of QPVA-Based Membrane in Passive Alkaline Direct Ethanol Fuel Cells

**DOI:** 10.1186/s11671-018-2836-3

**Published:** 2019-01-18

**Authors:** Z. Zakaria, S. K. Kamarudin, S. N. Timmiati

**Affiliations:** 1 Fuel Cell Institute, Universiti Kebangsaan Malaysia, 43600 Bangi, Selangor Malaysia; 2 Faculty of Engineering and Built Environment, Universiti Kebangsaan Malaysia, 43600 Bangi, Selangor Malaysia

**Keywords:** Passive alkaline–DEFCs, Graphene oxide, Quaternized poly (vinyl alcohol)

## Abstract

Passive alkaline–direct ethanol fuel cells (alkaline–DEFCs) appear to be suitable for producing sustainable energy for portable devices. However, ethanol crossover is a major challenge for passive alkaline–DEFC systems. This study investigated the performance of a crosslinked quaternized poly (vinyl alcohol)/graphene oxide (QPVA/GO) composite membrane to reduce ethanol permeability, leading in enhancement of passive alkaline–DEFC performance. The chemical and physical structure, morphology, ethanol uptake and permeability, ion exchange capacity, water uptake, and ionic conductivity of the composite membranes were characterized and measured to evaluate their applicability in fuel cells. The transport properties of the membrane were affected by GO loading, with an optimal loading of 15 wt.% and doped with 1 M of KOH showing the lowest ethanol permeability (1.49 × 10^−7^ cm^2^ s^−1^ and 3.65 × 10^−7^ cm^2^ s^−1^ at 30 °C and 60 °C, respectively) and the highest ionic conductivity (1.74 × 10^−2^ S cm^−1^ and 6.24 × 10^−2^ S cm^−1^ at 30 °C and 60 °C, respectively). In the passive alkaline–DEFCs, the maximum power density was 9.1 mW cm^−2^, which is higher than commercial Nafion 117/KOH (7.68 mW cm^−2^) at 30 °C with a 2 M ethanol + 2 M KOH solution. For the 60 °C, the maximum power density of composite membrane achieved was 11.4 mW cm^−2^.

## Introduction

Fuel cell is an electrochemical device that converts the chemical energy of fuel directly into electric energy without any combustion. This technology is more efficient in providing electrical energy compared to the conventional method; 60% of fuel conversion able to achieved, while the conventional methods require several conversion steps to produce the electrical energy [1]. Among the various type of fuel cell, the direct ethanol fuel cell (DEFC) is a promising portable source of power because it offers easy fuel storage, has simple design, and use less toxic fuel than other types of fuel cell [2–4].

For portable power source device applications, passive DEFCs seems more suitable and reliable compared to active DEFCs due to the fuel delivery concept and easy handling. The passive feeding system employ the natural capillary force without involving any pump for fuel and air breathing to supply oxygen for the redox reactions. Hence, no additional power consumption is required. The compact and light structure of a single cell can be developed for a compact device, which is different from active cells that require an external pump and blower, thus enlarging the cell [5–7]. Unfortunately, two disadvantages of passive DEFCs impede their commercial use: the sluggish electro-kinetic reactions of the anode and the high ethanol permeability from anode to the cathode [8]. Currently, Nafion® is used in DEFCs and also used in other types of fuel cell in acidic and alkaline medium due to its high proton conductivity and high mechanical stability. However, the Nafion® membrane has several drawbacks that hinder DEFC commercialization, including a poor ethanol barrier, high production cost (higher than U.S Department of Energy target of ~ 40$/m^2^), and the use of harmful materials in its production. The high ethanol permeability of DEFCs results in power loss as well as cathode catalyst contamination [8–10].

To address the passive DEFC problems stated above, the passive alkaline direct ethanol fuel cells (alkaline–DEFCs) were explored by researchers as alternative system. The passive alkaline–DEFCs work in alkaline conditions to achieve the following advantages over the acidic condition systems: (1) the rate of ethanol oxidation in the anode catalyst is higher, (2) the cost of using non-platinum catalysts, such as Ag or Ni, is lower, and (3) the OH^−^ flow direction is opposite the ethanol diffusion flow in the membrane and thus can significantly reduce ethanol crossover of the membrane due to the reduced influence of ionic motion [11–13]. As the “heart of the DEFC”, the use of anion exchange membranes has been investigated widely in recent years [8]. Commercial quaternary ammonium AEMs can conduct hydroxide anions; unfortunately, the conductivity of AEMs is still poor. In addition, the functional groups in conventional AEMs are necessary in the higher concentration alkaline fuel consumption and degraded when the operational temperatures exceed 60 °C [14–17]. Therefore, an alternative membrane for AEMs is urgently needed for passive alkaline–DEFC applications.

Poly (vinyl alcohol) (PVA) is an attractive polymer which has potential to replace the commercial AEMs due to its excellent chemical resistance, mechanical stability, and ability to serve as a fuel barrier. Additionally, preparing PVA membranes is simple because the film formation process is very easy, and the hydrophilic properties of its hydroxyl groups has facilitate the high density of reaction sites for crosslinking reactions, which improve the mechanical properties and thermal stability of the membrane [18–21]. The maximum power density of PVA-based membrane can reach until 8.0 mW cm^−2^ under ambient conditions for the passive mode of alkaline–DEFCs and 82 mW cm^−2^ at 80 °C for the active mode of alkaline–DEFCs as reported in previous studies [4, 13].

In alkaline media, PVA-doped potassium hydroxide (KOH) makes PVA as an effective stable electrolyte, which form the hydrogen bonds and dipole-dipole interactions that result in good ionic conductivity in the stable structure of AEMs [22–25]. Based on previous studies, KOH-doped PVA-based membranes tolerate a Fenton solution (e.g., a redox environment) and demonstrate significant chemical resistance [23, 26]. Hence, this combination has higher chemical stability toward alkaline treatment than other anionic exchange membranes. When nanofillers are integrated into the PVA matrix, a physical crosslinking mechanism can further improve the dimensional stability and resist the dissolution in water and ethanol crossover. Moreover, PVA is an inexpensive material that reduces the cost of DEFCs fabrication and it is nontoxic and biodegradable, making it as a green material [11, 23, 27, 28].

Unfortunately, there are two major drawbacks of PVA if applied as AEM, including poor ionic conductivity and high swelling ratio due to high water uptake [29, 30]. Introduction of quaternary ammonium functional group into the PVA matrix plays an important role in enhancement of ionic conductivity. Quaternized poly (vinyl alcohol) (QPVA) is derived from the modification of PVA with glycidyltrimethyl-ammonium chloride (GTMAC). QPVA resolves the problem of low ionic conductivity as well as improving the hydrophilicity and water selectivity [31–34]. Xiong et al. [31] reported that the ionic conductivity of QPVA membrane was 7.34 × 10^−3^ S cm^−1^ as AEM, while Yang et al. [34] has succeeded in enhancing the ionic conductivity of a QPVA-based membrane up to 2.11 × 10^−2^ S cm^−1^ at 60 °C. However, based on our knowledge, no work yet has been carried out to discover the potential of a QPVA-based membrane on a passive alkaline–DEFC system.

As reported in previous work, QPVA-based membrane has high water uptake, which leads to the high fuel permeability and decrease the mechanical strength. This condition can be improved through the blending process with an inorganic filler or other polymers to form a QPVA composite-based membrane [35, 36]. Rajesh Kumar et al. [37] reported that the ethanol permeability of a QPVA-based membrane was high, in the range of (0.95–2.08) × 10^−6^ cm^2^ s^−1^. In this study, QPVA is combined with graphene oxide (GO) as AEMs to enhance the performance of passive alkaline–DEFCs with low ethanol permeability and high ionic conductivity. Currently, GO is the most widely used inorganic material due to its special characteristics, including its potential to improve the thermal and mechanical strength of polymers, good networking ability due to the presence of rich functional. GO is a good candidate as a filler in polymer electrolyte due to the oxygenic functional groups, including hydroxyl, carboxylic, and epoxy groups, present at the edge of GO. This hydrophilic region is useful to transfer the anion across the polymer electrolyte [38–41]. Karim et al. [42] reported that the ionic conductivity of GO was nearly 10^−2^ S cm^−1^ higher than bulk of graphite oxide (10^−4^ S cm^−1^). In addition, the hydrophobic region in the aromatic ring (sp^2^ carbon layer) of the GO nanosheet structure is useful to reduce the fuel crossover issue and enhance the mechanical strength due to the strong covalent bonding [40–44]. Lin et al. [44] reported that GO sheet was able to decrease the fuel permeability of Nafion®115 membranes until 10^−7^ cm^2^ s^−1^. Ye et al. [45] applied the PVA/GO electrolyte membrane in an alkaline direct methanol fuel cell to enhance cell performance, which result in increment about 126% of ionic conductivity and 55% reduction in fuel permeability.

Apparently, there are no studies that have been reported before in investigating the performance of crosslinked QPVA/GO composite membranes in passive alkaline–DEFC systems. The objective of this study is to prepare a self-synthesized QPVA-based membrane with suitable nanofiller loadings that effectively decreases the ethanol crossover, thus enhancing the performance of the passive alkaline–DEFC. Homemade GO was dispersed in the QPVA polymer matrix during the solution casting method to enhance the membrane performance. The properties of the prepared crosslinked QPVA/GO composite membrane with different GO loadings and doped with 1 M KOH were evaluated by several tests such as liquid uptake, ion exchange capacity, ionic conductivity, and ethanol permeability. The optimal composition of GO was determined based on the best performance regarding on membrane selectivity measurements. The performance comparison between crosslinked QPVA/GO composite membranes and potassium hydroxide (KOH)-doped commercial Nafion 117 membrane (designed as Nafion 117/KOH) to provide the alkaline electrolyte membrane in passive alkaline–DEFCs has been examined, and it is useful as a guideline concerning in the application of alternative AEMs in passive alkaline–DEFCs.

## Methodology

### Materials

Poly (vinyl alcohol) (PVA) (Mw 85,000–124,000, 99 + % hydrolyzed), graphite powder, sodium nitrate (NaNO_3_), potassium permanganate (KMnO_4_), and glycidyltrimethylammonium chloride (GTMAC) were supplied by Sigma Aldrich. Glutaraldehyde (GA, 25% in distilled water) was provided by Nacalai Tesque, Japan. Phosphoric acid (H_3_PO_4_), sulfuric acid (H_2_SO_4_), hydrochloric acid (HCl), sodium hydroxide (NaOH), and potassium hydroxide (KOH) were purchased from J.T. Baker, and hydrogen peroxide (H_2_O_2_) was purchased from HmbG Chemical. All of these commercial materials were used without any purification.

### Synthesis of Quaternized PVA

An appropriate amount of PVA was dissolved in deionized water while stirring with a magnetic stirrer at 90 °C for 2 h. When the resulting PVA solution became homogeneous, transparent, and viscous, then the temperature of the solution was reduced to 65 °C. Next, the appropriate amounts of GTMAC and KOH (GTMAC:KOH = 1:1 mol ratio) were introduced, and the resulting homogeneous and colorless solution was continuously stirred for 4 h. Anhydrous ethanol was added to the solutions to obtain yellow precipitates of quaternized poly (vinyl alcohol) (QPVA), and then the precipitates were dried in a vacuum oven.

### Synthesis of Graphene Oxide

The Hummer’s method was used to synthesis graphene oxide (GO) [46]. Two grams of graphite were mixed with 2 g of NaNO_3_ in a volumetric flask (500 mL). Then, 150 mL of H_2_SO_4_ was added to the mixture, and the mixture was continuously stirred for 30 min in an ice bath (0–5 °C). Next, 12 g of KMnO_4_ was added to the mixture, and the reaction temperature was maintained below 20 °C for 4 h. The ice bath was removed, and the reaction was stirred for 1 day at 35 °C until the solution appeared pasty and brown. Next, the solution was diluted with 100 mL of deionized (DI) water to form a brown solution. Then, 200 mL of water was added to reduce the temperature. Finally, 10 mL of H_2_O_2_ was poured to treat the solution, and a yellow color was observed. The centrifuge was used to purify the solution with 10% HCl, and rinsing with DI water was performed several times for the purification step.

### Preparation of the Crosslinked QPVA/GO Composite Membrane

**Fig. 1:**
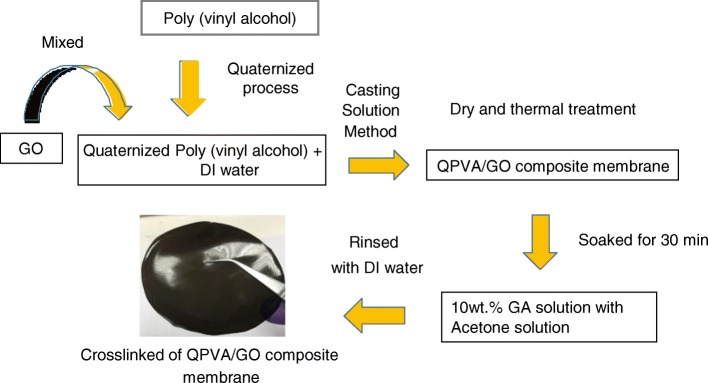
Schematic diagram of the preparation of QPVA/GO composite membrane

A schematic diagram of the preparation steps of the crosslinked QPVA/GO composite polymer membrane is shown in Fig. 1. QPVA (8 wt.%) was fully dissolved in DI water by magnetic stirring for 1 h at 75 °C to form a homogenous and transparent solution. Solutions with various GO contents (5 wt.% to 20 wt.%) were provided by diluting the GO solution (1 g/L). The solution of QPVA was continuously stirred while adding the GO solution (2:1 volume ratio) for 1 h to produce a yellowish-brown QPVA/GO solution. The yellowish-brown solution was continuously stirred with 10 wt.% GA for 30 min. The solution was cast onto a plastic plate and controlled the volume of solution to fix the thickness of membrane (0.015 mm). Then, dried under ambient conditions for 24 h and in a vacuum oven at 60 °C for 12 h. The membrane was peeled off from the plastic plate and placed on a glass plate for the annealing process at 100 °C for 1 h. Then, the membranes were immersed in 1 M KOH solution at 80 °C for 24 h. The excess KOH on the surface of the membrane was removed by repeatedly rinsing with DI water, and the membrane was stored in DI water at room temperature prior to use.

### Structural and Morphological Characterization of the Composite Membrane

#### Fourier Transformation Infrared Spectroscopy Analysis

Infrared absorption spectra of pristine PVA membrane, pristine QPVA membrane, and crosslinked QPVA/GO 15 wt.% composite membrane were recorded on a Perkin Elmer Fourier-transform infrared spectroscopy (FTIR) with an ATR (attenuated total reflection) module. The samples were held in a sample holder and placed in the ATR–FTIR system. The FTIR studies were conducted at ambient temperature, and the spectral range was 400–4000 cm^−1^.

#### X-Ray Diffraction

X-ray diffraction (XRD, model D8 Advance, Bruker AXS Germany) measurements were obtained from the crosslinked QPVA/GO composite membranes to examine their crystallinity. The X-ray radiation was generated using Cu Kα radiation (wavelength of 0.15406 nm) from an anode with an operating power of 40 kV and 40 mA. The scanning rate was 0.5°s^−1^, and the resolution was 0.02°. The XRD spectra were recorded from angles of 10° to 50°.

#### Field Emission Scanning Electron Microscope–Energy-Dispersive X-Ray Spectroscopy, Transmission Electron Microscopy, and Atomic Force Microscopy

The surface and cross-sectional morphologies of the crosslinked QPVA/GO composite polymer membrane were viewed using a ZEISS SUPRA 55 VP field emission scanning electron microscope (FESEM) at 10 kV. Energy-dispersive X-ray spectroscopy (EDX) was used for element mapping. Dry membrane was manually fractured after cooling in liquid nitrogen. Transmission electron microscopy (TEM-Hitachi HT 7700, Japan) and atomic force microscopy (AFM) were performed to show the presence and size of exfoliated GO.

### Thermogravimetric Analysis

The thermal properties of composite membrane were determined through thermogravimetric analysis (TGA). PVA membrane, QPVA membrane, and QPVA/GO composite membrane samples were heated from an ambient temperature to 600 °C under a nitrogen atmosphere in increments of 10 °C min^−1^ using a thermogravimetric analyzer (TGA Q500 V20.13 Build 39).

### Membrane Uptake, Swelling Ratio, Ion Exchange Capacity, and Oxidative Stability

The alkaline, water, and ethanol uptake of membrane were determined from the loss weight of the crosslinked QPVA/GO composite membrane before and after hydration. The alkaline uptake procedure was performed by immersing the membrane sample into 2 M KOH at 30 °C for 24 h. Then, the samples were removed from KOH, the surface humidity was removed from the samples with tissue paper, and the samples were weighed on a microbalance mass immediately to obtain the wetted membrane weight (*W*_wet_). Then, the samples were dried in a furnace at 100 °C for 2 h to determine the dried membrane weight (*W*_dry_). The alkaline uptake (*W*) was calculated using the following Eq. (1):

1 $$ W=\frac{W\ \mathrm{wet}-W\ \mathrm{dry}}{W\ \mathrm{dry}}\times 100 $$

For the water and ethanol uptake, the procedure was modified by replacing the solution treatment with water and 2 M ethanol.

The swelling ratio of the crosslinked QPVA/GO composite polymer membrane was examined by immersing the 100 mm^2^ samples in DI water at 25 °C for 24 h. Then, the sample was taken out from DI water, the surface humidity was removed with tissue paper, and the thickness of the membrane was measured to obtain the wetted membrane thickness (*t*_wet_) using a micrometer (Mitutoyo, ± 1 μm). Then, the samples were dried in a furnace at 100 °C for 2 h to determine the dried thickness (*t*_dry_). The swelling ratio (Sr _DI water_) was calculated using the following Eq. (2):

2 $$ Sr=\frac{t\ \mathrm{wet}-t\ \mathrm{dry}}{t\ \mathrm{dry}}\times 100 $$

The classical titration method was used to measure the ion exchange capacity (IEC) of the composite membranes. The composite membranes were soaked in a 0.1 M NaOH solution to convert them into OH^−^. Then, the composite membranes were washed with DI water to remove excess NaOH and equilibrated with 100 mL of 0.1 M HCl solution for 48 h. Next, the IEC values were determined from the reduction of acid measured using back titration. The formula used to calculate the IEC values (meq g^−1^) is given below:

3 $$ \mathrm{IEC}\kern0.37em \left(\mathrm{meq}.{\mathrm{g}}^{\hbox{-} 1}\right)\kern0.5em =\kern0.75em \frac{M_{\mathrm{b}}-{M}_{\mathrm{a}}}{m\ } $$

where *M*_b_ represents the milliequivalents (meq) of HCl required before equilibrium, *M*_a_ represents the HCl required after equilibrium, and *m* is the mass (g) of the dried composite membrane.

The oxidative stability test was measured via Fenton’s reagent (3% H_2_O_2_ aqueous solution containing 2 ppm FeSO_4_). The samples (40 mm × 40 mm) were immersed in the Fenton’s reagent at 25 °C. The weight of membrane before and after immersion was recorded after 24 h. Any changes of membrane as sample start to break or melt in the solution was the notice of its maximum time of test.

### Ionic Conductivity, Ethanol Permeability, and Selectivity Factor

The ionic conductivity was determined using a four-electrode conductivity cell connected to an impedance analyzer potentiostat/galvanostat (WonATech-WMPG1000), which was used to obtain the resistance of the membrane composites from the slope of the E–I curve [47]. All measurements were carried out using Eq. (4):

4 $$ \sigma =\frac{L}{RS} $$

where σ, *L*, *R*, and *S* represent the proton conductivity (σ, S cm^−1^), the distance between the counter electrodes (L, cm), the resistance of the membranes (R, S^−1^), and the cross-sectional area of the membrane samples (S, cm^2^), respectively. The crosslinked PVA/GO composite membranes were equilibrated in deionized water at various temperatures (30–60 °C). The membranes were positioned in the transverse direction and sandwiched between the electrodes.

A diffusion cell that contained two glass compartments was built to determine the membrane ethanol permeability. The two compartment glasses were divided to form the feed compartment, which was filled with 2 M, 4 M, 6 M, or 8 M ethanol, and a second chamber which filled with deionized water. Each compartment contained a magnetic stirring bar for solution agitation. The membrane was clamped vertically between the two glass compartments [47]. During the experiment, the concentration of ethanol that crossed the membrane was measured. The membrane permeability was calculated using the following Eq. (5):

5 $$ P=\frac{1}{Ca}\left(\frac{\Delta  Cb(t)}{\Delta  t}\right)\left(\frac{LVb}{A}\right) $$

where *P* represents the ethanol diffusion permeability of the membrane (cm^2^ s^−1^), Ca represents the concentration of the feeding chamber in cell A (mol L^−1^), *∆Cb*(*t*)/*∆t* represents the slope of the molar variations of the ethanol concentration in cell B as a function of time (mol L^−1^ s), Vb represent the volume in each of the diffusion reservoirs (cm^3^), *A* represent the membrane, and *L* represent the thickness of the membrane (cm). All the concentration solution was measured with refractometer. The selectivity factor of the crosslinked QPVA/GO composite polymer membranes (the ratio of the ionic conductivity to the ethanol permeability) was determined using the following Eqs. (6) and (7):

Selectivity,

6 $$ \Phi =\frac{P}{\sigma } $$

### Single Cell of Passive Alkaline–DEFC Performance and Durability Test

**Fig. 2:**
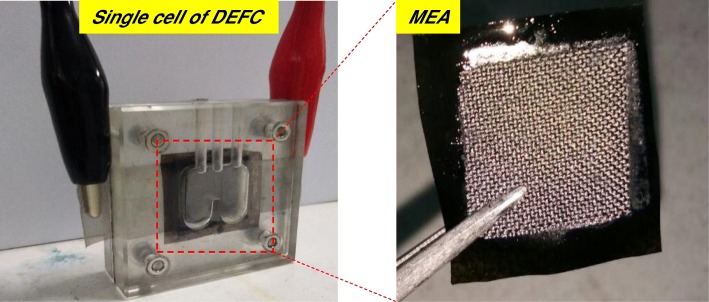
The single cell of passive alkaline–DEFC and MEA

Figure 2 shows the single cell of passive alkaline–DEFC homemade and the membrane-electrode assembly (MEA). The MEA for the passive alkaline–DEFC test cell was constructed, and the composite membrane was sandwiched between both electrodes (the anode and cathode) via a hot-pressing machine. For the anode, a Pt–Ru catalyst was applied at 4 mg cm^−2^, and for the cathode, a Pt catalyst was applied at 4 mg cm^−2^ to a carbon paper gas diffusion layer via a manual casting technique. Pt–Ru and Pt were supplied by Alfa Aesar, USA. The DEFC performance was tested through passive air-breathing with a homemade single-cell stack with an active MEA area of 4 cm^2^. The fuel reservoir region of the single cell stack allows for 2 mL of fuel. The polarization data were obtained using the potentiostat/galvanostat (WonATech) to measure the voltage response by applying the load current to a single cell under ambient conditions. Next, 2 M KOH + 2 M ethanol was reacted as fuel in the anodic reservoir, and the surrounding air diffused into the cathode opening during the performance testing. The single cell of passive alkaline–DEFC was examined to a durability test for a period of 1000 h at 60 °C. Due to passive feeding fuel, the ethanol solution will need refueling every 12 h. The durability performance testing was conducted in continuous operation constant load with the cell voltage was constant at 0.3 V. After the durability test finished, the polarization test of single cell was repeated to compare the performance.

## Results and Discussion

### Characterization and Morphology of the Composite Membranes

**Fig. 3:**
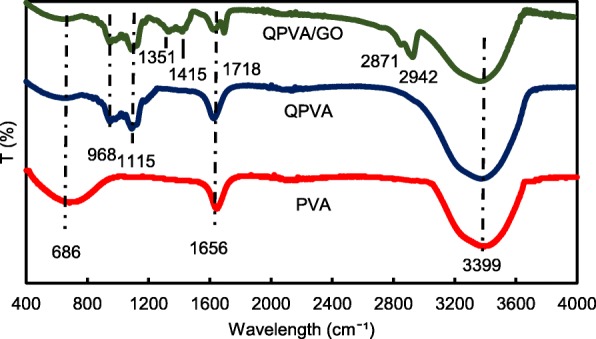
FTIR spectra with wavelength 400–4000 cm^−1^

The degree of quaternization is 2.6% and the remaining 97.4% is matrix polymer of PVA [23]. Six different type of membranes were produced; pristine PVA membrane, crosslinked QPVA membrane, and four of crosslinked QPVA/GO composite membranes with different weight percentage, 5 wt.%, 10 wt.%, 15 wt.%, and 20 wt.%, respectively. Table 1 lists the result of the elemental mapping of the pristine PVA membrane and the crosslinked QPVA/GO 15 wt.% composite membrane by EDX spectroscopy. The PVA composite membrane contained carbon (C) and oxygen (O). Meanwhile, the crosslinked QPVA/GO 15 wt.% composite membranes contained carbon (C), nitrogen (N), and oxygen (O), indicating that the quaternary ammonium group was successfully grafted into the PVA matrix. Introducing GO into QPVA caused an increase in weight and the atomic percentage of O inside the composite membrane due to the oxygenic functional groups from GO (hydroxyl, carboxyl, and epoxy groups). The evidence supporting the engagement of QPVA and GO were further examined using vibrational spectroscopy from FTIR analysis. The functional groups of PVA, QPVA, and QPVA/GO 15 wt.% composite membranes were recorded in the region of 400–4000 cm^−1^, as shown in Fig. 3.

**Table 1:** The results of element mapping of the PVA membrane and QPVA/GO 15 wt.% composite membrane

Membrane	PVA		QPVA/GO 15 wt.%	
Element	Wt.%	At. %	Wt.%	At. %
C	66.62	72.54	49.60	44.27
N	0	0	5.28	6.26
O	33.0	27.46	45.12	39.47

Figure 3 shows the wavelength range from 400 to 4000 cm^−1^. The absorption peak at 686 cm^−1^ in the PVA membrane corresponds to the aliphatic C–H bending [3]. After the quaternization and crosslinked process, aliphatic C–H bending was reduced, and several new peaks appeared in the spectrum of the crosslinked QPVA membrane and the crosslinked QPVA/GO 15 wt.% composite membrane. The peak at 968 cm^−1^ was characterized as aliphatic C-N, indicating that the quaternary ammonium group was grafted onto the backbone of the matrix polymer [31]; and the peak at 1115 cm^−1^ indicate the C–O–C stretching group that appeared after the crosslinking treatment and proves that the crosslinking reaction occurred and that the composite membrane was successfully crosslinked by GA [12]. There are several new peaks that appear in the composite membrane compared to pristine PVA and crosslinked QPVA; the peak at 1351 cm^−1^ represents epoxy C-O symmetric vibrations from GO [23]; the peak at 1415 cm^−1^ represents the stretching vibrations of carboxylic groups (O=C-O) from GO [44]; the deformation of the peak at 1656 cm^−1^ attributed to the integrated of C=C stretching from the aromatic structure corresponding to the sp^2^ character of the GO hydrophobic region and reaction of backbone of matrix polymer [39]; the new peak at 1718 cm^−1^ resulted from -COOH stretching in the carboxylic group in the GO [23]; the new peaks appear in the composite membrane at 2871 cm^−1^, which belong to =C–H stretching and at 2942 cm^−1^, the increase in the asymmetrical and symmetrical stretching of –CH_2_ from the GO can be observed. After the modification of PVA, a strong and broad absorption at 3399 cm^−1^ can be observed due to the weakening of –OH stretching affected by the formation of hydrogen bonds with the functional group of GO. From the FTIR spectral analysis, the peaks of the quaternization process of PVA and the synthesis of GO clearly demonstrated functional groups and hydrophobic regions similar to previous studies [23, 31, 44], which indicate that the crosslinked QPVA/GO composite membrane was successfully synthesized.

**Fig. 4:**
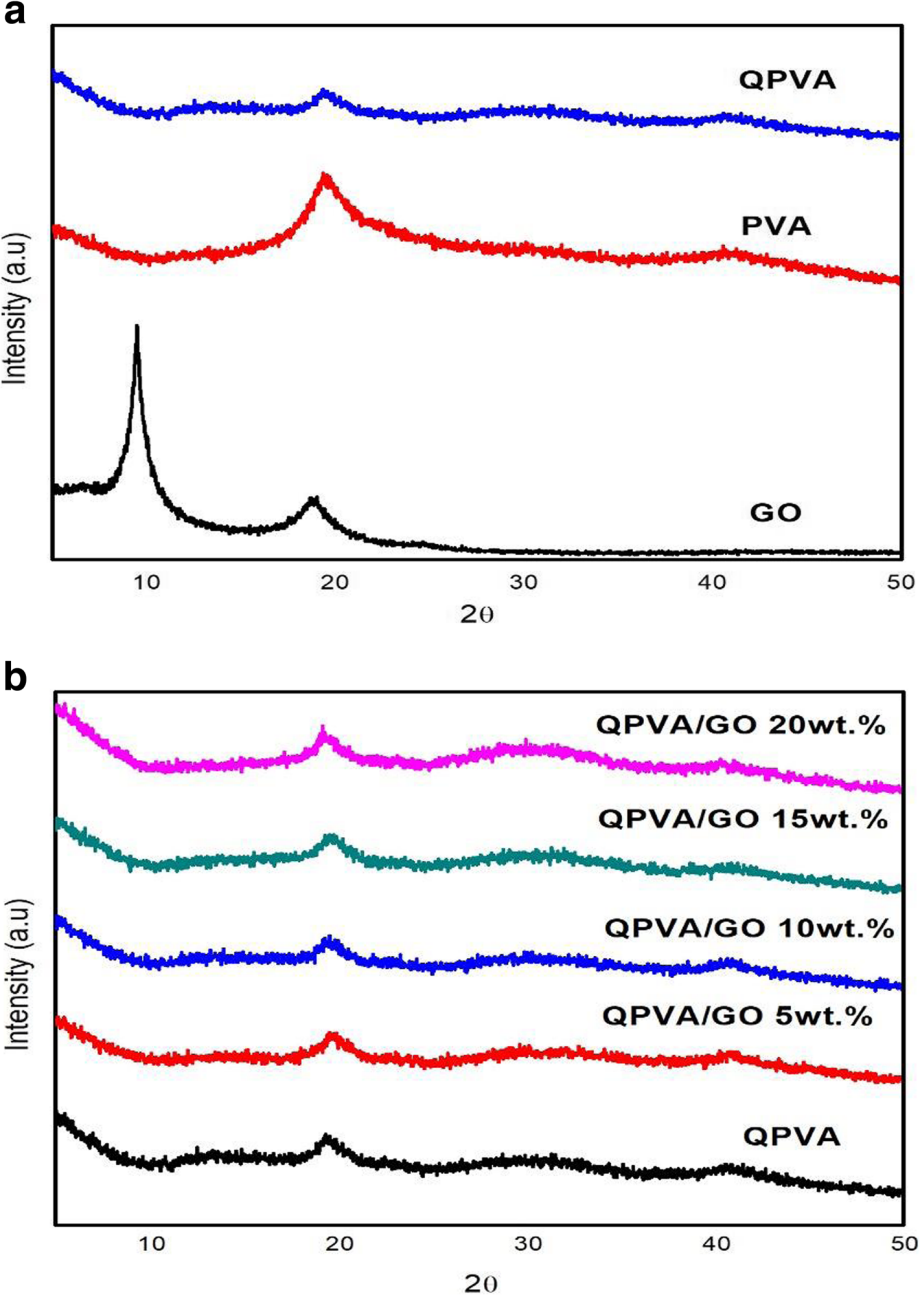
XRD patterns of **a** GO, PVA, and QPVA; **b** QPVA, QPVA/GO composite membrane

X-ray diffraction measurement was performed to observe the crystallinity of the composite membrane. The illustrations of the diffraction pattern for the XRD analysis peak are presented in Fig. 4a, b. Lee et al. [43] has reported that the peak of graphite is at 25.6°. Figure 4a shows the XRD spectra of homemade GO with the progressive phase change from graphite to GO. The peak intensity is slightly higher at 10.92° due to exfoliated of GO after oxidation process. The changes occur due to the interaction between hydrophobic region with the dispersed oxygenic functional group at the edge of GO benzene structure [43]. The large peak presence in the XRD pattern at 19–20° and the small peak at 39–40° were indicators of the semi-crystalline structure of PVA as shown in Fig. 4a. The peak intensity of QPVA at 19–20° was decreased compared to the pure PVA due to the introduction of ammonium functional group that disturbed the PVA semi-crystalline structure. Hence, the amorphous region becomes domain in the QPVA polymer matrix. This structure has contributed in the ionic conductivity enhancement due to the increasing of free volumes in the polymer chain which provide more free space which act as the ionic path through the membrane with the local structural relaxation and segmental motions of the polymer [26]. Figure 4b shows that the large diffraction peak of GO was invisible, which indicated that GO was homogenously dispersed in the QPVA matrix through physical interaction. Notably, the GO peak at 2θ = 10.92° was almost invisible due to the complete exfoliation and destruction of the original sheet by incorporation into the QPVA polymer. This result confirmed that the synthesis of a crosslinked QPVA/GO composite membrane involved the formation of hydrogen bonding and the interactions at the ionic level between the polymer chains and the functional groups in GO [23, 39]. The diffraction intensity of the QPVA at 19–20° was still detected, implying that the degree of QPVA crystallinity was maintained until the additional of graphene oxide at 20 wt.% loading. Hence, the chemical structure of the QPVA composite films barely changed as the graphene oxide loading increased, indicating that there were many physical interactions but few chemical reactions occurring between the QPVA and graphene oxide in the composite membrane formation process [39, 48]. In addition, the amorphous structure of composite membrane has significant effect in ionic conductivity due to the formation of free volume by constantly fragmental movement of the polymer matrix. Thus, the presence of GA as a crosslinked and KOH as a doped inside the polymer matrix provided more pathway for ionic to pass through the membrane [27, 49–51].

**Fig. 5:**
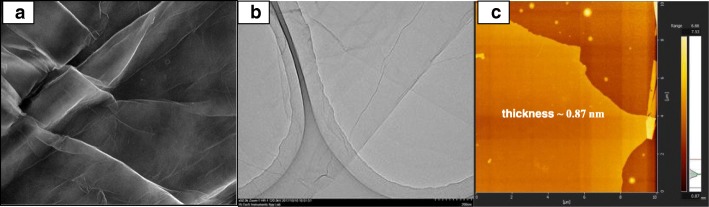
FESEM, TEM, and AFM image of exfoliated graphene oxide

**Fig. 6:**
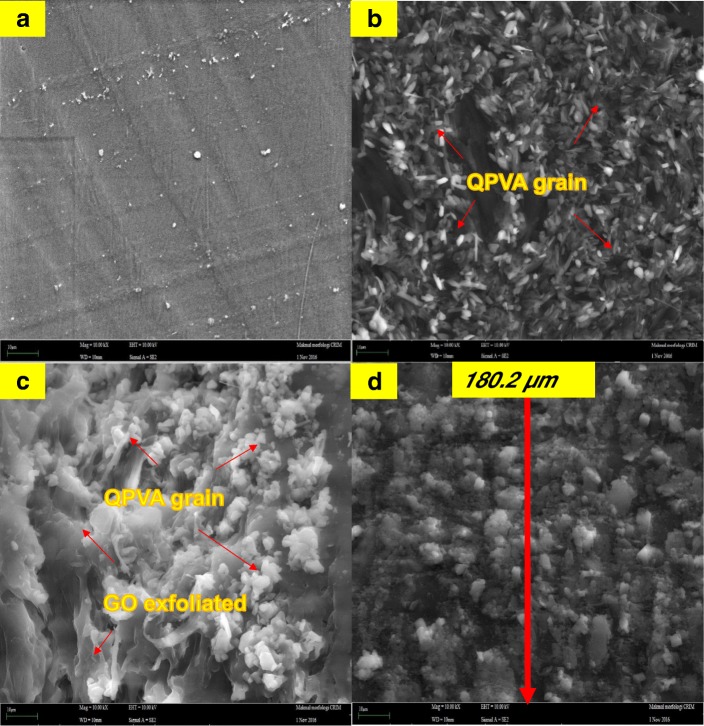
FESEM image of **a** surface PVA membrane, **b** surface QPVA membrane, **c** surface of QPVA/GO composite membrane, and **d** cross section of QPVA/GO composite membrane

The FESEM, TEM, and AFM images were used to identify the morphological features of GO as shown in Fig. 5. Figure 5a, b shows the FESEM and TEM images which clearly seems that the single layer of graphene oxide (likes a paper sheet) was successfully exfoliated from the graphite flakes. AFM was used to measure GO layer thickness of 0.87 nm as presented in Fig. 5c. The GO thickness obtained has confirm the exfoliated single layer of GO has successfully synthesized. The morphologies of the prepared membrane are shown in Fig. 6a–d by FESEM at a magnification of × 10.00 k. All the top view of FESEM images show that all membranes are dense and remain uncrack, although there are annealing in 100 °C, which proves the excellent chemical stability due to the cross-linkage and modification with GO in QPVA matrix. This characteristic is important for using as polymer electrolyte membrane in DEFC applications, especially to prevent the fuel crossover. The surface of the PVA membrane seems very smooth as shown in Fig. 6a, while the QPVA membrane in Fig. 6b shows a different morphology, as the grain distribution obviously appeared, indicating that the PVA was successfully grafted with quaternary ammonium groups [27]. Figure 6c shows the top view of the QPVA/GO composite membrane, indicating the random distribution of GO inside the QPVA which proved that GO has successfully exfoliated and dispersed throughout the membrane. From the cross-sectional view in Fig. 6d, the composite membrane exhibit a dense and compact structure with high homogeneity, indicating the unification of QPVA and GO in the composite membrane without any holes. In addition, the presence of GA and KOH filled the free volume inside the polymer matrix. This is the important characteristics for separation and fuel crossover reduction. Hence, the homogeneous exfoliation of GO, dispersion of GA and KOH improve the ionic conductivity and reduce the ethanol permeability [23, 24, 52, 53].

### Thermal Stability

**Fig. 7:**
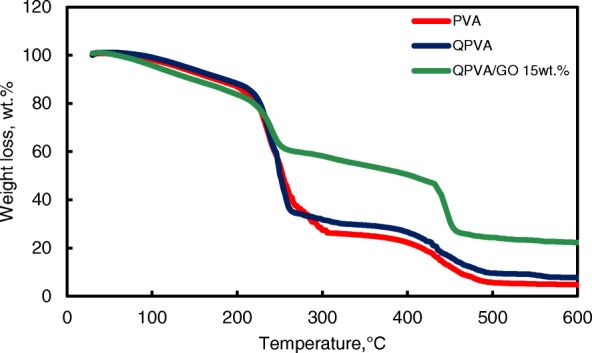
TGA analysis of PVA membrane, QPVA membrane and QPVA/GO composite membrane

The thermal stability of the crosslinked QPVA/GO composite membrane was illustrated by TGA studies. Figure 7 shows the TGA curves for the pure PVA membrane, QPVA membrane, and QPVA/GO 15 wt.% composite membrane. The TGA curves of these three membranes present three major weight loss regions. The first region is at 70–130 °C due to the loss of adsorbed water in the membrane through the evaporation of the weak and strong physical and chemical bonds of water, respectively. The weight loss of all membranes is approximately 5–8 wt.%. The second transition region of weight loss at approximately 230–320 °C is ascribed to the decomposition of the side chains of the matrix polymers. The weight loss of the membrane was 75 wt.% for PVA, 70 wt.% for QPVA, and 60 wt.% for QPVA/GO; the total weight loss of PVA was the highest due to the presence of fewer side chain groups compared to QPVA/GO. The third transition region of weight loss at approximately 430–480 °C was ascribed to the decomposition of the main chain of the membrane due to cleavage of the C–C backbone of the matrix polymers. At 600 °C, the residual mass of the PVA membrane was 4.7 wt.%. The residual mass of the QPVA membrane and the QPVA/GO membrane were 7.67 wt.% and 22.2 wt.%, respectively.

Figure 7 shows that the weight loss of the QPVA/GO 15 wt.% membrane was less than the other membranes; this result indicates that the quaternization process of the PVA matrix and the incorporation of GO inside the composite membranes was successful. The inorganic filler enhanced the thermal stability of the composite membrane and produced a strong network between the matrix polymer and the GO filler reinforcement. In addition, the chemical crosslink reaction by glutaraldehyde assists in the enhancement of thermal stability.

### Alkaline Uptake, Swelling Ratio, and Oxidative Stability

Alkaline uptake is the major process for electrolyte preparation. The KOH used for alkaline doping serves as a charge carrier and cross-linker. The alkaline–DEFC was operated with alkaline anode fuel (alcohol in KOH solution). Alkaline uptake in the membrane is important for providing OH^−^ ions. The optimal KOH uptake condition is necessary to provide high ionic conductivity. Nevertheless, excessive KOH uptake leads to the deformation of the matrix polymer and degrades the mechanical stability of alkaline–DEFC by shortening its lifetime. The dry membrane films were immersed in 2 M KOH at 30 °C, and the dimensional stability was estimated using the changes of the membrane weight for alkaline uptake and the width, length, and thickness for the swelling ratio of the membrane films before and after doping with 2 M KOH. Table 2 shows that the alkaline uptake of the PVA membrane was 105%. After the quaternization process, the hydrophilic structure C–H and the presence of quaternary ammonium groups led to an alkaline uptake of QPVA membrane increase up to 136%. However, the introduction of GO in QPVA associated the reduction of alkaline uptake until to 45% with 20 wt.% loading of GO. The hydrophobic region of GO contributes a “blocking effect” in the polymer matrix and thus reduced the KOH absorption of the membranes. Furthermore, the crosslinking agent might also reduce the alkaline uptake due to the more compact network structures formed in the composite membranes [23, 50]. The alkaline uptake has influence the swelling behavior of the composite membranes.

**Table 2:** Water uptake and swelling ratio

Membranes	KOH uptakes (%)	Swelling ratio (%)		Oxidative stability (Retained weight, %)
		In-plane	Through-plane	
PVA	105	20	62.41	96
QPVA	136	18	60.75	97
QPVA/GO 5 wt.%	114	15.2	53.13	95
QPVA/GO 10 wt.%	98	12.3	42.54	94
QPVA/GO 15 wt.%	84	8.65	35.48	93
QPVA/GO 20 wt.%	45	7.54	28.5	91

The in-plane swelling (i.e., width and length) and through-plane swelling (i.e., thickness) ratios of the composite membranes are shown in Table 2. Generally, a low membrane swelling ratio is favorable for tolerating the dimensional match between the electrode and membrane; thus, the stable formation of MEA is encouraged during single cell performance. The in-plane and through-plane swelling ratios for the QPVA membrane were 18% and 60%, respectively. The introduction of GO up to 20 wt.% loading into QPVA reduced the in-plane and through-plane swelling ratios to 7.54% and 32%, respectively. The introduction of GO not only favors the formation of connected ionic transport channels but also expands the hydrophobic region of GO inside the matrix QPVA, which results in the dimensional stability of the composite membranes [23, 52]. The crosslinked QPVA/GO 20 wt.% composite membrane showed lower swelling ratios than the pristine QPVA membrane. Liao et al. [26] reported a low in-plane swelling ratio due to the strong interactions between the polymer matrix and filler, which involve dipole-dipole interactions, hydrogen bonding, and van der Waals interactions between the polymer and GO. All the samples exhibit good in-plane swelling ratios rather than through-plane swelling ratios, which are beneficial for the MEA contact conditions.

The oxidative stability of the anion exchange membrane was measured in the Fenton’s reagent test which is known as hastened oxidative stability test. Table 1 shows the retained weight percentage of the crosslinked QPVA/GO membranes. After 24-h immersion, the retained weight of the QPVA membrane is 97%. It was slightly declined with increasing of GO loading which retained weight of 91% for 20 wt.% of GO. The obtained results demonstrated that the free radicals have little impact on the QPVA-based membranes due to the greater oxidative resistivity of hydrogen bond formed between GO, GA, and polymer matrix. This may be due to the presence of oxygen contained functional groups present in the GO that easily forms hydrogen bonding with pristine QPVA [1, 23, 54]. The weight loss percentage results indicate that the crosslinked QPVA/GO membrane has good oxidative stability against Fenton’s reagent.

### Water Uptake, Ion Exchange Capacity, and Ionic Conductivity

**Fig. 8:**
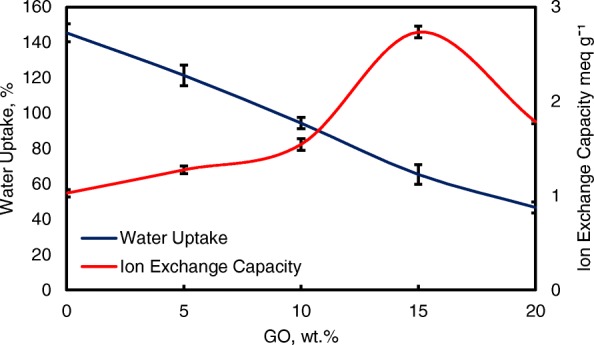
Water uptake and IEC analysis of QPVA membrane and QPVA/GO composite membrane at 30 °C

Water uptake is an important parameter for ionic transfer inside the electrolyte membrane. But, higher of water uptake will degrade the membrane performance [8, 50]. From the Fig. 8, the modification of QPVA-based membrane and GO has successfully reduce the water uptake form 145 to 46%. Ion exchange capacity (IEC) is an important parameter for evaluating the mobility ability of anion through the membrane. Besides, IEC is useful to determine the range of optimal water uptake which enhance the conductivity of membrane through the vehicle mechanism [33, 55]. Figure 8 shows that the values of the experimental IEC ranged from 1.05 to 2.71 meq g^−1^ according to the increase of GO loading. The existence of quaternary ammonium group grafts on matrix polymer and increasing amounts of GO has led to the addition of oxygenic functional groups, including hydroxyl, carboxyl, and epoxy groups, which are useful for anion transfer through the Hopping @ Grotthus mechanisms [22, 35]. Higher IEC values are the main indicator of higher ionic conductivity due to the high charge density inside the membrane [33, 55].

**Fig. 9:**
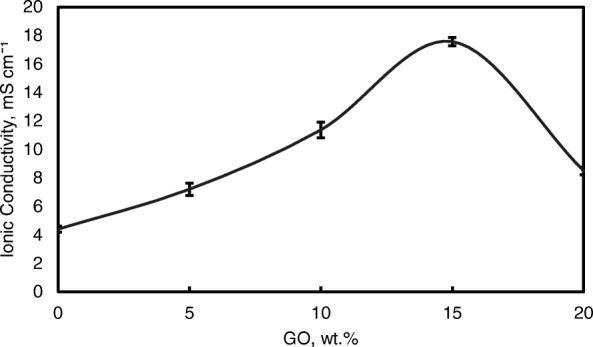
Ionic conductivity analysis of QPVA membrane and QPVA/GO composite membrane at 30 °C

Figure 9 also shows the ionic conductivities of the QPVA membrane and the crosslinked QPVA/GO composite membrane at 30 °C. The ionic conductivity of the QPVA membrane (4.7 × 10^−3^ S cm^−1^) is higher compared to the PVA membrane (1.05 × 10^−3^ S cm^−1^). The grafted quaternary ammonium group at the PVA backbone assists in increasing the ionic conductivity due to the OH^−^ that has been transferred effectively through the pathway created by the presence of functional groups in GO [31]. The ionic conductivity of composite membranes was increased which recorded as 7.3 × 10^−3^ S cm^−1^, 11.3 × 10^−3^ S cm^−1^, and 17.56 × 10^−3^ S cm^−1^ for GO loading at 5 wt.%, 10 wt.%, and 15 wt.%, respectively. The introduction of GO in the composite membrane up to 10 wt.% showed a slight increase in ionic conductivity attributed to the “blocking effect” of the GO sp^2^ characteristic reacting as a hydrophobic region for ionic clusters in the composite membrane.

**Fig. 10:**
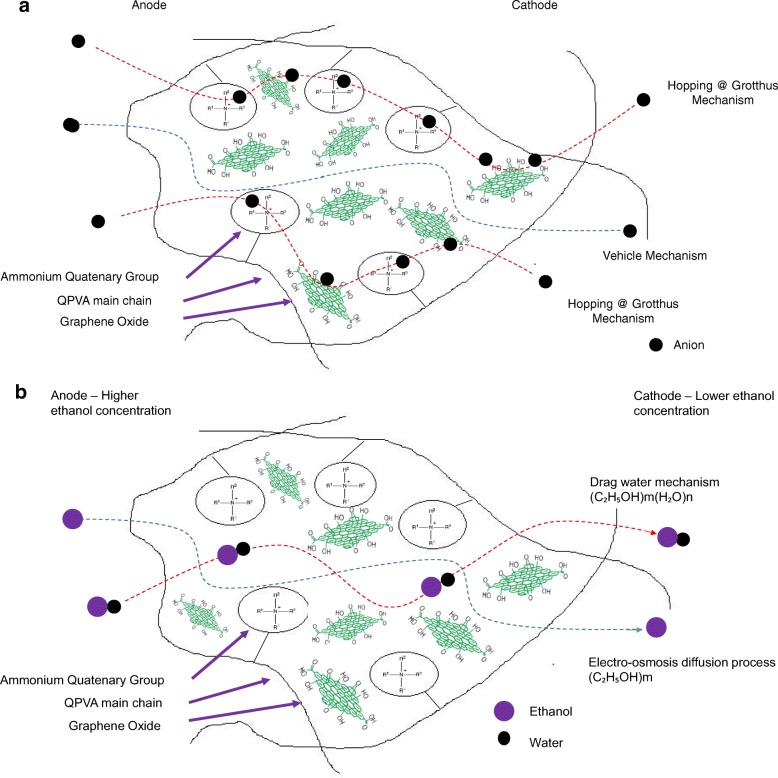
**a** Illustration of ionic transfer for the crosslinked QPVA/GO composite membrane. **b** Illustration of ethanol transfer for the crosslinked QPVA/GO composite membrane

However, beyond 10 wt.% of GO loading (15 wt.%), the ionic conductivity of the composite membrane increased to 17.35 × 10^−3^ S cm^−1^ due to the sufficiently highly oxygenated functional groups (hydroxyl, carboxyl, and epoxy) in GO to provide alternative anion pathways as well as overwhelming the blocking effect from the hydrophobic region. Thus, the addition of GO has enhance the ionic conductivity of the composite membranes [43]. The oxygenic functional groups of GO, such as carboxyl groups, can form complexes with KOH molecules and form good ionic conductors with high-speed channels for anion exchange and transport. This condition is useful for hopping mechanism [23, 52]. Moreover, the strong interactions between GO, the polymer matrix, and GA form a three-dimensional network that can hold water and alkaline molecules that are important in the Vehicle mechanism. Ionic transport depends on the alkaline molecules and water uptake occurs by the vehicle mechanism. In the composite membranes, the alkaline molecules could be classified as free alkaline and bound alkaline. For conductivity, only the free alkaline affects the anion transport [23, 56]. Figure 10a presents the ionic transport illustration for crosslinked QPVA/GO composite membrane.

**Fig. 11:**
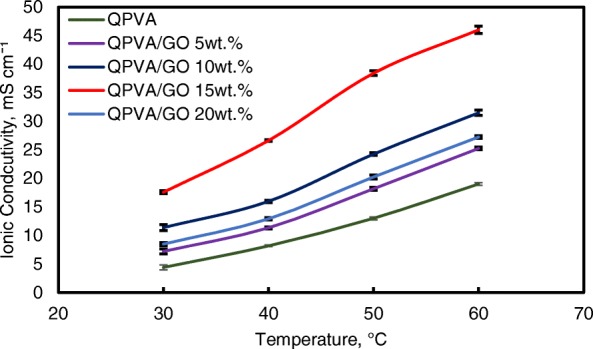
Ionic conductivity analysis of QPVA membrane and QPVA/GO composite membrane at different temperature

However, when the GO loading exceeded 20 wt.%, the ionic conductivity of the composite membrane declined due to the decrease in alkaline uptake by the composite membrane, which was attributed to the GO hydrophobic region. Hence, this condition disturbs the Grotthuss mechanism in conveying the ionic diffusion, thus reducing the ionic conductivity of the composite membrane. Higher temperature increases the ionic conductivity due to the effect of fast anion mobility. In addition, the distance between the polymer chains becomes larger at higher temperature, providing more free volume and facilitating water molecule movement to enhance anion conductivity [1, 45]. Figure 11 shows the trend of the ionic conductivity when the temperature increased. Increasing temperature also led to increasing ionic conductivity. The highest ionic conductivity was 4.6 × 10^−2^ S cm^−1^ at 60 °C for 15 wt.% GO loading. Compared to the previous study on PVA-based membranes in DEFCs, such as PVA/HPW/DPTA, PVA/TMAPS, PVA/LDH, and PVA/CNTs [11–13, 50], the current QPVA/GO membrane showed the highest ionic conductivities.

**Fig. 12:**
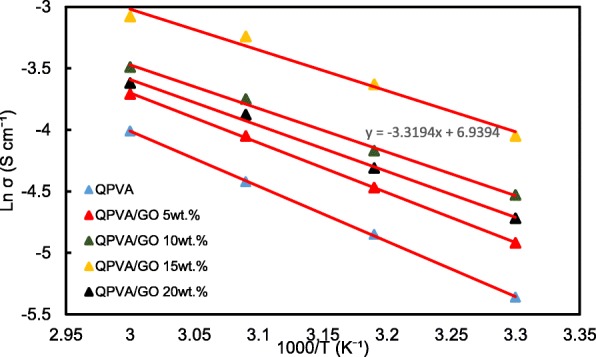
Ln σ vs. 1000/T plot for the QPVA/GO composite membrane; the lines indicate the linear regression

The ln σ and 1000/T plots are shown in Fig. 12 with the assumption that the ionic conductivity follows Arrhenius behavior. The activation energy *E*_*a*_ of the transferred ion in the composite membranes can be obtained according to the Arrhenius equation:

7 $$ {E}_a=-b\times R $$

where *b* is the slope of the regression line for the plotted graph (ln σ vs.1000/T) and *R* represents the gas constant (8.314472 J K^−1^ mol^−1^). The QPVA/GO 15 wt.% composite membrane has the lowest activation energy of 18.11 kJ mol^−1^ compared to the other composite membranes. The GO loading at 15 wt.% resulted in sufficient presence of functional groups in the composite membrane, also providing an optimal structure for the effective anion transport to subsequently contribute to the reduction of the activation energy.

### Ethanol Uptake and Ethanol Permeability

**Fig. 13:**
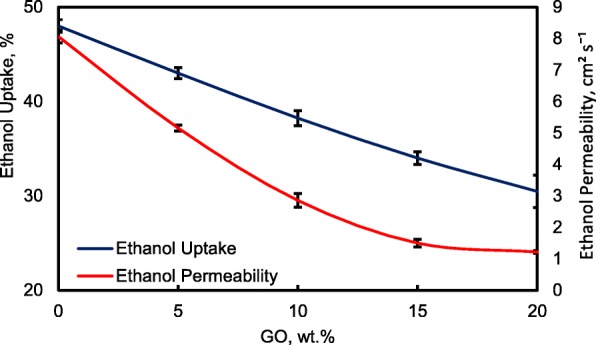
Ethanol uptakes and Ethanol permeability of QPVA membrane and QPVA/GO composite membrane at 30 °C

**Fig. 14:**
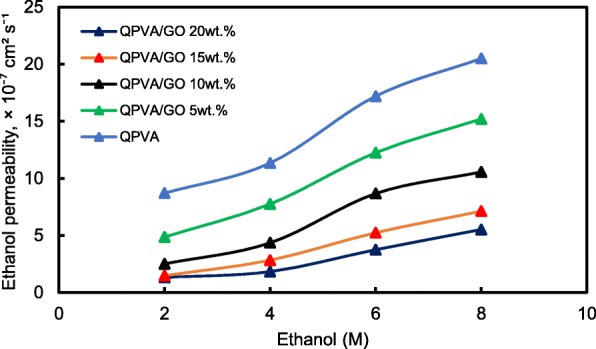
Ethanol permeability of QPVA membrane and QPVA/GO composite membrane with different ethanol concentration

Figure 13 shows the ethanol uptake and ethanol permeability of the pristine QPVA and the crosslinked QPVA/GO (5–20 wt.%) composite membrane in 2 M ethanol at 30 °C. One PVA property is its slight solubility in ethanol, which effectively reduces ethanol crossover [57]. The ethanol uptake clearly indicates that the QPVA polymer absorbs less ethanol than water. With 20 wt.% GO loading, ethanol uptake decreased by ~ 35% (from 52% by the pristine QPVA membrane to 34% by the crosslinked QPVA/GO 20 wt.% composite membrane). This behavior can be explained by the possible appearance of a three-dimensional network built by the crosslinking effect of GA on GO and the polymer matrix. These results are important because they can indicate the dimensional stability of the composite membranes under the set conditions and show their barrier properties and ethanol permeability. The reduction in ethanol uptake was mainly provided from the optimum GO loading, which increased the formation of a three-dimensional network between GA, GO, and PVA. Thus, the free volume of the polymer composite decreased and thus resisted the ethanol mobility pathway [12, 50, 55]. Figure 13 shows similar result of ethanol permeability for the pristine membrane and composite membranes. The ethanol permeability of the membrane decreased by ~ 85% (from 8.7 × 10^−7^ cm^2^ s^−1^ for the pristine membrane to 1.32 × 10^−7^ cm^2^ s^−1^ for the crosslinked QPVA/GO 20 wt.% composite membrane). The reduction of ethanol permeability is affected by increasing the GO content of the composite membrane. The three-dimensional network between GA, GO, and PVA formed a compact structure that increased the resistance of the membrane to ethanol crossover. Besides, the presence of KOH as electrolyte was fulfilled the free volume space in polymer matrix [24, 50, 55]. Figure 10b has illustrated the ethanol transport in crosslinked QPVA/GO composite membrane. The ethanol permeability of the composite membranes has also been studied in different ethanol concentrations (2, 4, 6, and 8 M) at 30 °C, as shown in Fig. 14. The result showed that the ethanol permeability is lower in order of range ~ 10^− 7^ cm^2^ s^−1^. The reduction in ethanol permeability was attributed to the presence of the hydrophobic region when the GO content increased, which functions as an ethanol crossover blockage [55, 58]. At the same temperature, the crosslinked QPVA/GO composite membrane exhibited lower ethanol permeability than the PVA/phosphotungstic acid solutions, with different percentages of diethylenetriaminepentaacetic acid. The ethanol permeability of the crosslinked QPVA/GO composite membrane is in the range of 1.32–8.7 × 10^−7^ cm^2^ s^−1^, which is lower than the previous study using QPVA with fume silica (14–16 × 10^−6^ cm^2^ s^−1^) in 3 M and 5 M ethanol [40]. The ethanol permeability increased with increasing in ethanol concentration. This result is the initial indicator that the open-circuit voltage of a single cell with this composite membrane will decrease with increasing ethanol concentration.

**Fig. 15:**
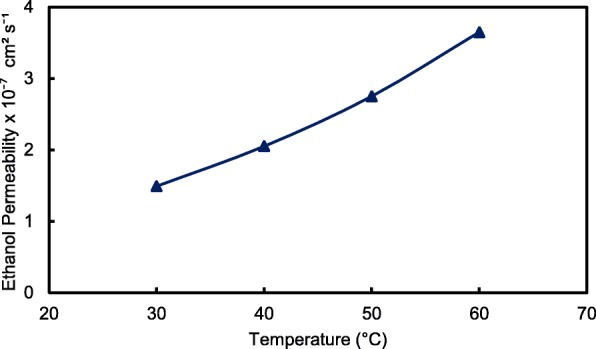
Ethanol permeability of QPVA/GO 15 wt.% composite membrane with different temperature

The crosslinked QPVA/GO composite membrane with the highest ionic conductivity (15 wt.% QPVA/GO) at 30 °C was particularly observed in the ethanol permeability test for various temperatures to assess its performance for portable devices in the range of 30 °C to 60 °C, as shown in Fig. 15. The higher temperature leads in higher ethanol permeability due to the interaction between the membrane chains become more active. In addition, the free volume inside the membranes started to extend, which reduced the resistance for ethanol crossover. The diffusion cell was measured in a steady state without any electrical current. When the composite membrane was applied in DEFCs, the practical ethanol permeability was lower due to the movement of anion transport opposite of the direction of ethanol crossover from the anode to the cathode [8, 52].

### Selectivity of the Membrane, the Passive Alkaline–DEFC Performance and Durability Test

**Fig. 16:**
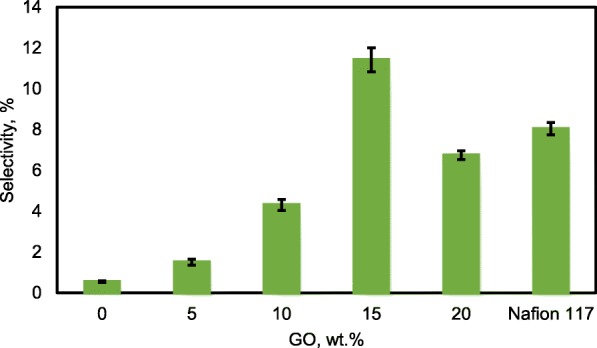
Selectivity of QPVA membrane, QPVA/GO composite membrane and Nafion 117

For the excellent single-cell application of passive alkaline–DEFCs, the membrane should have high ionic conductivity and low ethanol permeability. The ratio of both tests is called the selectivity factor. An excellent performance of a composite membrane is associated with a higher selectivity factor [55]. The selectivity of the composite membranes is presented in Fig. 16. The behavior was caused by the modification of QPVA and the introduction of GO with 15 wt.% loading due to the highest conductivity and the lowest ethanol permeability achieved. Therefore, this membrane has potential for further studies in single-cell alkaline–DEFCs. Table 3 shows a comparison between the ionic conductivity, ethanol permeability, and selectivity factor for PVA-based membranes in passive DEFC applications at 30 °C. The present study shows a comparable performance with the other previous works**.**

**Table 3:** Comparison of ethanol permeability, ionic conductivity, and selectivity factor for the PVA-based membrane in passive DEFC

Membrane	Types of membrane	Ethanol permeability, cm^2^ S^−1^	Ionic conductivity, S cm^−1^	Selectivity, S s cm^−3^	Power density, mW cm^−2^	Reference
Nafion®117	PEM	–	–	–	1.33	[5]
PVA/TiO_2_	AEM	2.81 × 10^−7^	0.048	17.08 × 10^4^	8.00	[14]
PVA/HPW/DTPA	PEM	2.39 × 10^−7^	0.0053	2.21 × 10^4^	–	[50]
PVA/GO	PEM	1.75 × 10^−7^	0.009	5.59 × 10^4^	5.84	[64]
PVA/HAP	AEM	–	0.044	–	10.74	[65]
QPVA	AEM	8.703 × 10^−7^	0.0073	0.83 × 10^4^	5.88	This study
QPVA/GO (30 °C)	AEM	1.48 × 10^−7^	0.017	11.48 × 10^4^	9.1	This study
QPVA/GO (60 °C)	AEM	3.65 × 10^−7^	0.0624	17.09 × 10^4^	11.4	This study
Nafion®117/KOH	AEM	1.15 × 10^−6^	0.089	7.69 × 10^4^	7.68	This study

**Fig. 17:**
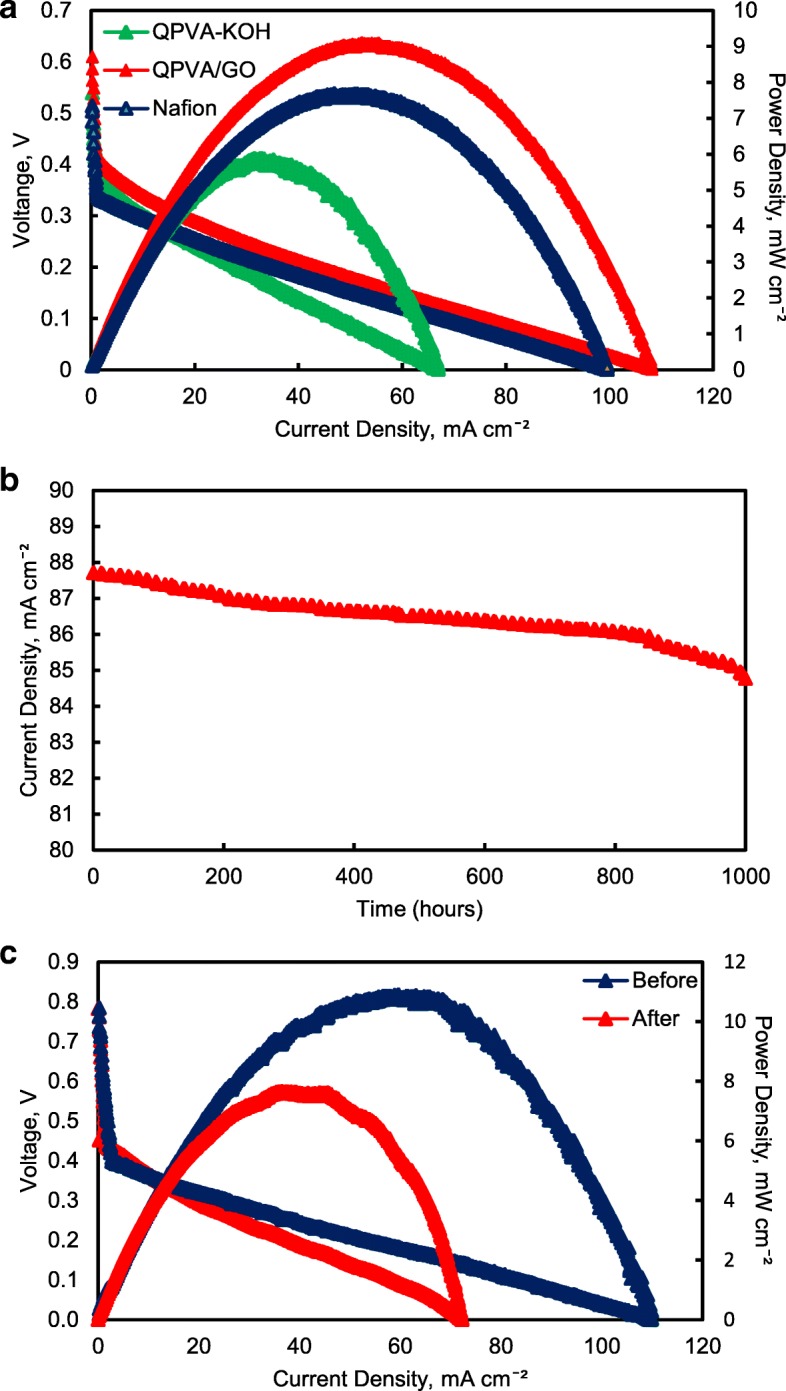
**a** Cell voltage and power density vs. current density curve obtained for the passive alkaline–DEFC. **b** Durability test on passive alkaline–DEFC. **c** Cell voltage and power density vs. current density curve before and after 10,000 min of durability test. Anode feed; 2 M KOH + 2 M ethanol, vathode feed; Air: **a** at 30 °C; **b**, **c** at 60 °C

Figure 17 illustrates the polarization and power density curves for passive alkaline–DEFCs at 30 °C for crosslinked QPVA, crosslinked QPVA/GO 15 wt.%, and Nafion 117 membranes. Obviously, the crosslinked QPVA/GO 15 wt.% composite membrane exhibited a higher open-circuit voltage (OCV) of 0.61 V, which can be attributed to the low ethanol permeability compared to QPVA and Nafion 117, which only reach OCV values of 0.54 V and 0.51 V, respectively. The OCV result was similar with previous study which used the platinum-based catalyst for the single cell test performance, in the range of 0.4 V to 0.6 V [15, 59, 60]. The lower concentration of fuel affected the OCV value, and Yuen et al. [61] reported for the best concentration for passive cell which used platinum catalyst based must be higher than 4 M to achieve the optimum cell performance. The maximum power density of the crosslinked QPVA/GO 15 wt.% composite membrane was 9.1 mW cm^−2^, which was higher than QPVA and Nafion 117 of 5.88 mW cm^−2^ and 7.68 mW cm^−2^, respectively. These results prove that the interaction between QPVA and GO occurred and was powerful to improve the potential of these membranes.

The performance of cell current density throughout the durability test operation at 60 °C was shown in Fig. 17b. The passive alkaline–DEFC was operated for 1000 h at a constant voltage of 0.3 V continuously. The current density of cells start with 87.7 mA cm^−2^ and was decreased continuous slowly until 1000 h operation and finally reaching approximately 84.7 mA cm^−2^ at 1000 h. This performance decreased steadily may be caused by the increasing of ethanol permeability which attributed the enhancement of fuel crossover without oxidation activity, which was significant negative effect on single cell performance [62]. The durability test results show high efficiency operation of crosslinked QPVA/GO 15 wt.% composite membrane in single cell which the sustainable performance until 1000 h.

Figure 17c shows the change of performance in cell voltage and power density of the crosslinked QPVA/GO 15 wt.% composite membrane before and after durability testing to evaluate the permanent degradation of alkaline passive–DEFC. When the operation temperature of single cell increase 60 °C, the OCV of cell increase to 0.78 V and the maximum power density of the crosslinked QPVA/GO 15 wt.% composite membrane increase 11.4 mW cm^−2^. After the durability testing, the maximum power density reduced to 7.65 mW cm^−2^. The dropped of power density was subjected to activation loss of catalyst activity due to ethanol crossover and the loss of maximum power density was 32.8%. The power loss depended on the current density drawn from the single cell. However, the value of OCV after the 1000 h test presented no significant reduction compared to before durability test [63].

Table 3 presents a comparison of PVA-based membranes previous study applied in passive DEFCs using a Pt-based catalyst. The performance in this study was higher than that of the passive DEFCs reported by Yang et al. [14] (8 mW cm^−2^) and higher than the optimization of commercial Nafion®117 membranes in passive DEFCs reported by Pereira et al. [5] (1.33 mW cm^−2^). This result demonstrated the success of our study targeting the performance of passive alkaline–DEFCs using crosslinked QPVA/GO composite membrane as alternative membrane for commercial membrane.

## Conclusion

New composite membrane was prepared by blending the QPVA polymer as a matrix and GO as a filler using GA as a crosslinking agent. The existence of a quaternary ammonium group grafted on PVA and modified with GO was confirmed by FTIR, XRD, and FESEM-EDX analysis. With the formation of three-dimensional networking between the polymer matrix, GO, and the crosslinking agent, the thermal stability of the composite membrane was enhanced. The highest ionic conductivity achieved was 0.046 S cm^−1^, along with a low ethanol permeability of 1.48 × 10^7^ cm^2^ s^−1^. The maximum power density of 9.1 mW cm^−2^ was observed for the crosslinked QPVA/GO 15 wt.% composite membrane at 30 °C and 11.4 mW cm^−2^ at 60 °C. Therefore, the crosslinked QPVA/GO composite membrane has high potential for use in single-cell DEFC applications. The process of optimizing the composition of QPVA/GO and the replacement of the non-Pt catalyst are expected to further enhance the performance of the QPVA/GO composite membrane.

## Abbreviations

A: Membrane
AFM: Atomic force microscopy
C: Carbon
Ca: Concentration of the feeding chamber in cell A
DEFC: Ethanol fuel cell
EDX: Energy-dispersive X-ray spectroscopy
FESEM: Field emission scanning electron microscope
GO: Graphene oxide
GTMAC: Glycidyltrimethyl-ammonium chloride
H_2_O_2_: Hydrogen peroxide
H_2_SO_4_: Sulfuric acid
H_3_PO_4_: Phosphoric acid
HCL: Hydrochloric acid,
KOH: Potassium hydroxide
L: Distance between the counter electrodes
L: Thickness of the membrane
m: Mass (g) of the dried composite membrane.
M_a_: HCl required after equilibrium
M_b_: HCl required before equilibrium
N: Nitrogen
NaOH: Sodium hydroxide
O: Oxygen
P: Ethanol diffusion permeability of the membrane
PVA: Poly (vinyl alcohol)
QPVA/GO quaternized: Poly (vinyl alcohol)/graphene oxide
R: Resistance of the membranes
*t*_wet_: Thickness
TEM: Transmission electron microscopy
TGA: Thermogravimetric analysis
Vb: Volume in each of the diffusion reservoirs
XRD: X-ray diffraction
σ: Proton conductivity
